# Serotyping, Genotyping and Virulence Genes Characterization of *Pasteurella multocida* and *Mannheimia haemolytica* Isolates Recovered from Pneumonic Cattle Calves in North Upper Egypt

**DOI:** 10.3390/vetsci7040174

**Published:** 2020-11-10

**Authors:** Ahmed H. Abed, Fawzy R. El-Seedy, Hany M. Hassan, Ashraf M. Nabih, Eman Khalifa, Salwa E. Salem, Gamal Wareth, Ahmed M. S. Menshawy

**Affiliations:** 1 Bacteriology, Mycology and Immunology Department, Faculty of Veterinary Medicine, Beni-Suef University, Beni Suef 62511, Egypt; frseedy@yahoo.com; 2 Animal Reproduction Research Institute, Giza 12511, Egypt; hanyhassan55@hotmail.com (H.M.H.); ashraf_nabih27@yahoo.com (A.M.N.); dr.salwasalem@hotmail.com (S.E.S.); 3 Microbiology Department, Faculty of Veterinary Medicine, Matrouh University, Matrouh 51511, Egypt; khalifa.eman@alexu.edu.eg; 4 Friedrich-Loeffler-Institut, Institute of Bacterial Infections and Zoonoses, Naumburger Str. 96a, 07743 Jena, Germany; gamal.wareth@fli.de; 5 Faculty of Veterinary Medicine, Benha University, Moshtohor, Toukh 13736, Egypt; 6 Veterinary Medicine Department (Infectious Diseases), Faculty of Veterinary Medicine, Beni-Suef University, Beni Suef 62511, Egypt; ahmed.elmenshawy@vet.bsu.edu.eg or

**Keywords:** *Pasteurella multocida*, *Mannheimia haemolytica*, serotyping, genotyping, virulence genes, calf pneumonia

## Abstract

*Pasteurella* (*P*.) *multocida* and *Mannheimia* (*M.*) *haemolytica* are the most two common pathogenic bacterial agents causing pneumonia in calves. Both bacteria are associated with significant economic losses in the cattle industry due to high morbidity and mortality rates, especially in the case of severe infections. The objectives of the present study were to perform serotyping and genotyping, as well as characterization of the virulence-associated genes in 48 bacterial isolates; 33 *P. multocida* and 15 *M. haemolytica*. All strains were isolated from pneumonic cattle calves showing respiratory manifestations such as fever, nasal discharges, and rapid breathing in North Upper Egypt governorates (Beni-Suef and El-Fayoum). PCR was applied as a confirmatory test using a specific universal gene, *kmt*1, and *rpt*2 for *P. multocida* and *M. haemolytica*, respectively. The results show that 29 (87.9%) *P. multocida* and 15 (100%) *M. haemolytica* isolates were positive for the corresponding universal gene. The results of serotyping indicate that 86.2% of *P. multocida* isolates belonged to serotype B:2, while 13.8% were untyped. Meanwhile, 60% and 40% of *M. haemolytica* isolates belonged to serotype 2 and serotype 1, respectively. Investigation of virulence-associated genes showed that all the tested *P. multocida* isolates harbored *nan*B, *omp*87, and *tox*A genes. Four *M. haemolytica* isolates harbored both *gcp* and *lkt*C genes and of these, three isolates harbored the *ssa* gene. Sequencing of *tox*A gene of *P. multocida* and *lkt*C gene of *M. haemolytica* in the current strains indicated a great homology with strains uploaded in gene banks from different hosts and localities worldwide.

## 1. Introduction

Bovine respiratory disease (BRD) is a significant cause of morbidity and mortality among beef cattle globally. *Pasteurella multocida* (*P. multocida*) and *Mannheimia haemolytica* (*M. haemolytica*), which are commensal Gram-negative bacteria in the upper respiratory tract of animals, represent the major bacterial causative agents for BRD [1]. Under stresses including environmental, managemental, and/or infectious factors, those agents often produce mild to severe clinical signs. The incubation period varies from 3 to 5 days. In peracute cases, sudden death within 24–36 h with clear clinical signs may be observed. In chronic cases, they may cause permanent lung damage such as fibrosis, adhesions and/or abscesses that are affecting the performance [1]. The clinical manifestations include a rise in temperature, respiratory distress with nasal discharge, and frothing from the mouth, and then recumbency and death may be the result [2]. Disease incidence is most often in 6–24 month-old animals and groups of less than 10 animals. The disease was seasonal, occurring only in rainy seasons of the year, and victims were only cattle and buffaloes [2]. Thus, early recognition and treatment of BRD are so important [3]. Respiratory disorders in animal production sectors in Egypt were reported to cause a considerable economic loss due to lower productivity and death [4]. *Pasteurella multocida* is a zoonotic bacterium causing hemorrhagic septicaemia (HS), which is a major disease of cattle and buffaloes occurring as catastrophic epizootics in many African and Asian countries characterized by an acute, highly fatal septicemia with high morbidity and mortality [3]. In humans, *P. multocida* has been tightly associated with animal exposure, usually involving soft-tissue sites within 24 h after animal bites, especially dog or cat, or scratch wounds. Serious respiratory tract infections including pneumonia, empyema, and lung abscesses are typically found in patients with underlying pulmonary disease. In more serious cases, a bacteremia can occur by spreading from a localized bite wound or from another localized source of infection, such as pneumonia, meningitis, or arthritis. Varieties of other serious invasive infections such as meningitis, endocarditis, and peritonitis, have also been reported, but are rare [5].

HS is caused by certain serotypes of *P. multocida* including B:2 in Asia and E:2 in Africa based on the Carter and Heddleston system, which is corresponding to the newer B:6 and E:6 serotypes in Namioka–Carter classification [6]. OIE added that A:1 and A:3 serotypes have been incriminated in an HS-like syndrome in bovines in India with mainly characterized by pneumonia ended with death. On the other hand, *M. haemolytica* (formerly *Pasteurella haemolytica*) is considered one of the most important pathogens in ruminants of all ages and it is the principal cause of bovine and ovine pneumonic pasteurellosis or shipping fever. It is responsible for considerable economic losses to the livestock industries all over the world [3,7]. Two biotypes have been recognized for the taxon *Pasteurella haemolytica*: biotype A, an isolate that ferments L-arabinose, and biotype T, an isolate that ferments trehalose [8]. Based on capsular polysaccharide antigen typing using the indirect haemagglutination test, *P. haemolytica* complex has been identified as 17 serotypes; including 13 A serotypes (A:1, A:2, A:5, A:6, A:7, A:8, A:9, A:11, A:12, A:13, A:14, A:16, and A:17) and 4 T serotypes (serotypes 3, 4, 10 and 15) [8]. *P. haemolytica* biotype A was later allocated to a new genus *Mannheimia* and renamed as *M. haemolytica*, while the four T serotypes were named *Bibersteinia trehalosi*. Later, serotype A:11 was classified into a new taxon as *M. glucosida*, due to its different biochemical profile, leaving 12 serotypes of *M. haemolytica* [9].

Many virulence genes are important in the pathogenesis of *P. multocida*, such as fimbriae and adhesins (*nan*B and *nan*H), a variety of outer membrane proteins (OMPs) such as protectins (*omp*A, *omp*H, *omp*87 and *plp*B), and toxins such as dermonecrotoxin (*tox*A) [1,10,11]. These virulence factors facilitate the colonization and invasion of *P. multocida* through impairing the host defense mechanisms, destruction of host tissues, and/or stimulation of a noxious host inflammatory response [12]. The *tox*A and the OMPs-encoding genes have been suggested as epidemiological markers as they are found in high prevalence in pneumonic *P. multocida* isolates [13]. *M. haemolytica* can colonize and establish infection in the lungs due to various virulence factors, including capsule, adhesins, lipopolysaccharide (LPS), OMPs, and various proteases [14]. The virulence of *M. haemolytica* is linked to different virulence genes, such as leukotoxin (*lkt*), leukotoxin C (*lkt*C), putative adhesin (*ahs*), O-sialoglycoprotease (*gcp*), outer membrane lipoprotein (*gs*60), transferring-binding protein B (*tbp*B) and UDP-N-acetyl-D-glucosamine-2-epimerase (*nma*A). Characterization of these genes provides important information about the pathogenicity of *M. haemolytica* [14,15]

The present study was designed to perform serotyping and genotyping of *P. multocida* and *M. haemolytica* isolates obtained from pneumonic calves in North Upper Egypt Governorates, as well as characterization of virulence-associated genes in both bacteria.

## 2. Materials and Methods

### 2.1. Sampling and Bacterial Isolation and Identification

A total of 189 pneumonic cattle calves of both sexes aged from 3 to 18 months were sampled. All calves showed respiratory manifestations including fever, nasal discharges, and rapid breathing during veterinary convoys in different villages and towns of North Upper Egypt Governorates (EL-Fayoum and Beni-Suef) during the period from January 2017 to December 2017. Forty-eight bacterial isolates (33 *P. multocida* and 15 *M. haemolytica*) were recovered from deep nasal swaps. Isolates were identified by traditional methods including morphology using Gram’s and Leishman’s stains, as well as colonial and biochemical characteristics. The suspected isolates of *P. multocida* and *M. haemolytica* were tested for hemolysis on blood agar and growth on MacConkey’s agar in addition to biochemical tests, i.e., oxidase, catalase, indole, triple sugar iron agar medium, citrate utilization and sugar fermentation (glucose, lactose, sucrose, and mannitol) tests [7]. The distribution of samples and bacterial isolates is shown in Table 1.

Before sampling, approval was obtained from the Ethical Committee at the Office of the Dean at faculty of veterinary medicine, Beni-Suef university (code, BSU/0139/24122016), as well as obtaining permission from the farm owners.

### 2.2. Molecular Confirmation and Serotyping of P. multocida and M. haemolytica Isolates by PCR

A PCR assay was applied on *P. multocida* and *M. haemolytica* isolates as a confirmatory test using specific universal genes for *P. multocida* (*kmt*1 gene) and *M. haemolytica* (*rpt*2 gene). Genomic DNA was extracted by QIAamp^®^ DNA extraction Mini Kit (Cat. No. 51304 supplied from QIAGEN, Valencia, CA, USA) according to manufacturer’s instructions. Extracted DNA was kept at −80 °C until being used in PCR amplification. The sets of the primer pairs were designed in Metabion Company (Planegg, Germany). Primers sequences and amplified products for the targeted genes for *P. multocida* and *M. haemolytica* isolates are illustrated in Table 2. Temperature and time conditions of the primers during PCR are shown in Table 3 according to specific authors and Emerald Amp GT PCR master-mix (Takara) kit.

All PCR-positive *P. multocida* and *M. haemolytica* isolates for *kmt*1 and *rpt*2 universal genes, respectively, were serotyped. *P. multocida* isolate serotyping was conducted using capsular type B antisera by rapid slide agglutination test according to Rimler and Rhoades [16]. Meanwhile, *M. haemolytica* isolates were serotyped using rapid plate agglutination procedure as described by Frank and Wessman [17]. Serotyping of *P. multocida and M. haemolytica* isolates was performed in the Department of Clinical Microbiology, Central Health Laboratories, Ministry of Health, Cairo, Egypt.

### 2.3. Detection of Virulence-Associated Genes in P. multocida and M. haemolytica Isolates by PCR

Detection of virulence-associated genes was done by PCR on ten multidrug-resistant (MDR) isolates (5 *P. multocida* and 5 *M. haemolytica*) which were selected according to the results of El-Seedy et al. [7]. The selected isolates were previously tested against 12 antimicrobial agents and showed resistance to oxytetracycline, ampicillin-sulbactam, amoxicillin-clavulanic acid, kanamycin, cefquinome, amikacin, ceftriaxone, ciprofloxacin, and enrofloxacin [7]. PCR was applied to determine the *nan*B, *omp*87 and *tox*A virulence-associated genes in *P. multocida* isolates and *ssa*, *gcp* and *lkt*C virulence-associated genes in *M. haemolytica* isolates. Primers sequences and amplified products for the targeted genes for *P. multocida and M. haemolytica* isolates are illustrated in Table 2. The temperature and time conditions of the primers during PCR are shown in Table 3.

### 2.4. Gene Sequencing and Sequence Analysis

Due to limited resources and funding, sequencing of *tox*A gene of one strain of *P. multocida* serotype B:2 as well as *lkt*C gene of one strain of *M. haemolytica* serotype 2 was applied. The amplified products of the *tox*A gene of *P. multocida* and *lkt*C gene of *M. haemolytica* isolates were purified from the gel using QIAquick Gel Extraction Kits (Qiagen Inc., Valencia, CA, USA). All steps of purification were run in accordance with the manufacturer’s instructions using reagents provided in the kits. For gene sequencing, the Bigdye Terminator V3.1 cycle sequencing kit (Perkin-Elmer/Applied Biosystems, Foster City, CA, USA, Cat. No.4336817) was used. The purified PCR products were sequenced along with sequencing primers (forward and reverse) on an applied biosystem 3130 automated DNA sequencer (ABI, 3130, USA). ABLAST^®^ analysis (basic local alignment search tool) was initially performed to establish sequence identity to gene bank accessions according to Altschul et al. [24]. The sequence reaction was done according to the instruction of the manufacture. A comparative analysis of sequence was performed using the CLUSTAL W multiple sequence alignment program, version 1.83 of the MegAlign module of laser gene DNA star software pairwise, which was designed by Thompson et al. [25]. Sequence alignments and phylogenetic analyses were done using maximum likelihood, neighbor-joining, and maximum parsimony in MEGA6 [26]. The sequenced genes of *P. multocida* and *M. haemolytica* Egyptian strains were compared with 14 and 17 strains, respectively, which were uploaded in gene bank representing different clinical lesions, hosts, and localities worldwide.

## 3. Results

### 3.1. Molecular Confirmation and Serotyping of P. multocida and M. haemolytica Isolates by PCR

The results of the PCR assay reveal that twenty-nine (87.9%) *P. multocida* isolates and fifteen (100%) *M. haemolytica* isolates were positive for the *kmt*1 and *rpt*2 universal genes, respectively (Table 4). Serotyping of *kmt*1 PCR-positive *P. multocida* isolates (*n* = 29) indicated that twenty-five isolates (86.2%) were *P. multocida* serotype B:2, while four isolates (13.8%) were untyped. Meanwhile, serotyping of *rpt*2 PCR-positive *M. haemolytica* isolates (*n* = 15) revealed that nine isolates (60%) were *M. haemolytica* serotype 2 (A:2) and six isolates (40%) were serotype 1 (A:1).

### 3.2. Detection of Selected Virulence-Associated Genes of P. multocida and M. haemolytica Isolates

Among five tested MDR *P. multocida* isolates, all harbored *nan*B, *omp*87, and *tox*A genes. Meanwhile, out of the five tested MDR *M. haemolytica* isolates, four isolates (80%) harbored both *gcp* and *lkt*C, of which three isolates (60%) harbored the *ssa* gene (Table 5).

### 3.3. Sequencing Analysis of the Selected Genes

**Figure 1:**
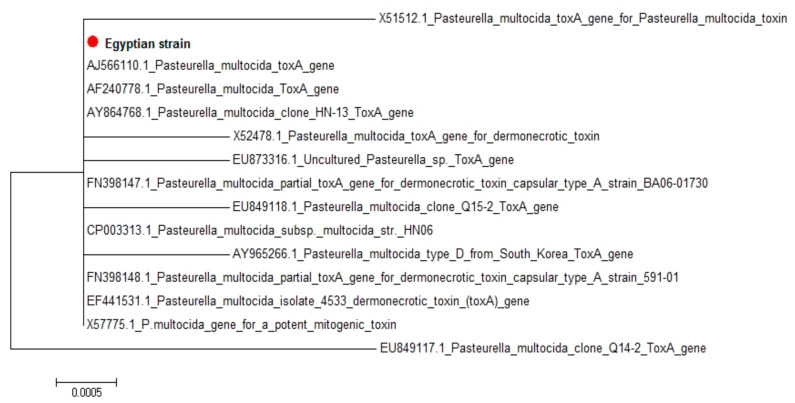
Phylogenetic tree for *P. multocida* serotype B:2 toxA showing clear clustering of the Egyptian strain with different strains uploaded in the gene bank.

Sequencing analysis of *P. multocida* serotype B:2 *tox*A gene: Amino acid and nucleotide alignment reports of the sequenced 276 amino acids and 828 bp nucleotides of *P. multocida* serotype B2 *tox*A showed great homology between the Egyptian strain and the different *P. multocida* strains uploaded in the genebank. The sequence distance of *P. multocida* serotype B:2 *tox*A was created by the MegAlign module of Lasergene DNAStar. Sequence identities between the isolated Egyptian strain and fourteen *P. multocida* strains uploaded in gene bank showed 99.4–100% homology (Table 6). The phylogenetic tree for *P. multocida* serotype B:2 *tox*A partial sequences was generated using maximum likelihood, neighbor-joining, and maximum parsimony in MEGA6. The phylogenetic tree showed clear clustering of the Egyptian strain with different *P. multocida* strains uploaded in the gene bank (Figure 1).

**Figure 2:**
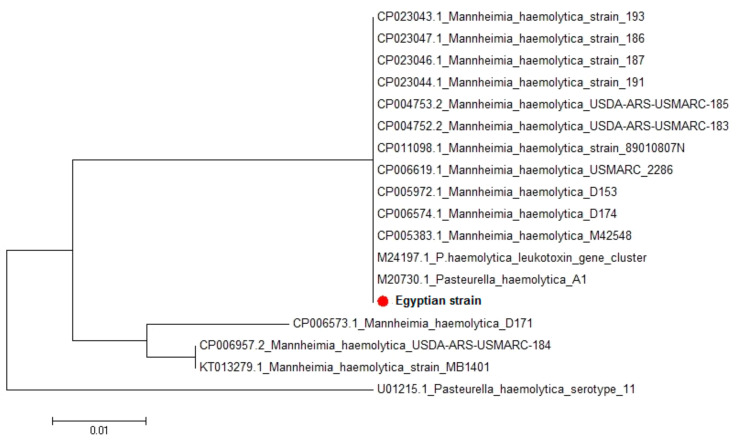
Phylogenetic tree for *M. haemolytica* A2 *lkt*C partial sequences showing clear clustering of the Egyptian strain with different strains uploaded in the genebank.

Sequencing analysis of *M. haemolytica* serotype 2 *lkt*C gene: Amino acid and nucleotide alignment reports of the sequenced 149 amino acids and 447 bp nucleotides of *M. haemolytica* A2 *lkt*C revealed great homology between the Egyptian strain and the different *M. haemolytica* strains uploaded in the gene bank. The sequence distance of *M. haemolytica* A2 *lkt*C was created by the MegAlign module of Lasergene DNAStar. Sequence identities between the isolated Egyptian strain and seventeen *M. haemolytica* strains uploaded in genebank showed 99.6–100% homology (Table 7). The phylogenetic tree for *M. haemolytica* serotype A2 *lkt*C gene partial sequences was generated using maximum likelihood, neighbor-joining, and maximum parsimony in MEGA6. The phylogenetic tree revealed clear clustering of the Egyptian strain with different *M. haemolytica* strains uploaded in genebank (Figure 2).

## 4. Discussion

Bacterial infections causing pneumonia in calves can be fatal. The pathogens *P. multocida* and *M. haemolytica* are the two most common bacterial agents causing calves’ pneumonia in Egypt and worldwide [7]. Respiratory disorders in animal production units in Egypt were reported to cause a considerable loss due to lower productivity and death [4]. One of the challenges of bovine respiratory medicine is the early recognition and treatment of clinical cases of BRD. As *P. multocida* and *M. haemolytica* are commensals in the upper respiratory tract of animals, they represent the main bacterial etiology for BRD. Successful treatment occurs if antibiotics were given at the initial stages of the disease [27]. *Pasteurella multocida*-polymerase chain reaction (PM-PCR) and capsular PCR assays are highly specific rapid efficient tools for the diagnosis of *Pasteurella* species, especially in epidemiological studies [28,29]. Capsular PCR assay was found to be a very convenient and reliable method for serogrouping of *P. multocida*, in contrast to the conventional serogrouping method, which is very slow and requires the production and maintenance of a battery of hyperimmune sera, which is difficult to produce [29,30]. Primers for *P. multocida* were designed to detect a fragment of the *kmt*1 gene encoding the outer membrane protein, producing an amplification product unique to all strains of *P. multocida* [31]. The present PCR method was designed to identify *P. multocida* serogroup B strains by amplification of the *kmt*1 gene with genotyping of it depending on the cap loci at *bcb*D that is highly specific for serogroup B.

The present study focused on molecular identification as well as serotyping and genotyping of *P. multocida and M. haemolytica* isolates from pneumonic calves in North Upper Egypt governorates. The PCR assay was applied as a confirmatory identification of *P. multocida* and *M. haemolytica* isolates using the *kmt*1 and *rpt*2 universal genes, respectively. The results of PCR reveal that 87.9% and 100% of *P. multocida* isolates and *M. haemolytica* isolates, respectively, were positive for the corresponding universal gene. The PCR results of *P. multocida* using the *kmt*1 gene are in agreement with the results obtained by Balakrishnan and Roy [32], who identified *P. multocida* strains recovered from sheep using the primers *kmt*1. In addition, Abbas et al. [31] identified the *P. multocida* isolates from different hosts using the primers *kmt*1 and they also considered these primers unique to all strains of *P. multocida*. Meanwhile, the obtained results for the detection of the *rpt*2 gene in *M. haemolytica* isolates are similar to those previously studied by Ryan and Lo [33]. Serotyping of *P. multocida* and *M. haemolytica* was conducted only on the PCR-positive isolates for universal genes. The results of the serotyping of *P. multocida* isolates (*n = 29*) using capsular type B antisera indicate that 86.2% isolates were serotype B:2, while 13.8% of isolates were untyped. These results run hand in hand with reports by Elshemey and Abd-Elrahman [2], who serotyped fifty *P. multocida* isolates from HS outbreak in cattle and buffalo in Alexandria province and found *P. multocida* type B:2 in 100% of strains. In addition, Abbas et al. [31] reported that 100% of *P. multocida* isolates from HS cases of cattle and buffalo in different governorates of Egypt belonged to serotype B:2. Regarding the untyped *P. multocida* isolates, after capsular and somatic antigen detection, some of the isolates cannot be differentiated because they may react similarly in both the antigens [34]. Furthermore, the agglutination of homologous antiserum may fail [31]. Passive hemagglutination has a substantial concern, as that test can be rendered ineffective by the loss of *P. multocida* capsule after repeated subcultures in vitro [35]. Moreover, the agglutination failure of serogroups A, D, and F with homologous antisera is one of the main causes of reduced sensitivity in this phenotypic test [36]. We speculated that the untyped isolates might be related to serogroups other than B, especially capsular group E, which is the most common in Africa. This point is supported by what was reported by OIE [6] and Farooq et al. [27], in that serotypes B:2 and E:2 were the most common serotypes of *P. multocida* associated with HS in animals in Asia and Africa, respectively.

The serotyping of *M. haemolytica* isolates (*n = 15*) showed that 60% and 40% of isolates belonged to serotype 2 and serotype 1, respectively. These results were supported by other studies [15,37,38], in which *M. haemolytica* serotypes (A:1, A:2, and A:6) were the most prevalent isolates recovered from cattle with BRD. Singh et al. [14] and Kabeta et al. [39] reported that HS is mainly caused by *M. haemolytica* serotype 1, and the disease is most commonly found in calves. Apart from outer membrane proteins and capsular antigens, the virulence-associated genes (*tox*A, *nan*B, *oma*87, and others) are playing important roles in the pathogenesis of *P. multocida* [40]. These virulence factors facilitate the colonization and invasion of *P. multocida* through impairing the host defense mechanisms, destruction of host tissues, and/or stimulation of a noxious host inflammatory response [12]. The *tox*A and the OMPs-encoding genes have been suggested as epidemiological markers and are found in high prevalence in pneumonic *P. multocida* isolates [13]. PCR-based methods have been used to ascertain their distribution in strains recovered from a wide range of sources and disease conditions [40]. The virulence of *M. haemolytica* is linked to different virulence genes including *lkt*, especially *lkt*C, *gcp*, and other genes, and characterization of these genes provides important information about the pathogenicity of *M. haemolytica* [14,15].

In the current study, PCR assay was applied on five MDR *P. multocida* isolates to determine *nan*B, *omp*87, and *tox*A virulence genes and on five *M. haemolytica* isolates to determine *ssa*, *gcp*, and *lkt*C virulence genes. The results indicate that all the tested *P. multocida* isolates harbored all the tested genes (100%); meanwhile, four of the tested *M. haemolytica* isolates (80%) harbored both *gcp* and *lkt*C, of which three isolates only (60%) harbored *ssa* gene. The obtained PCR results of *P. multocida* using *the tox*A gene agreed with data obtained by Devi et al. [41], who concluded that the *tox*A gene is an important marker gene for defining the pathogenic potential of *P. multocida* strains. Meanwhile, Ewers et al. [40] recorded that the *tox*A was found only in 12.5% of all isolates from small ruminants in Germany. In addition, Vougidou et al. [13] reported that some of the genes including the *tox*A and the OMPs-encoding genes have been suggested as epidemiological markers. On the contrary, Sarangi et al. [19] reported that all the virulence-associated genes, except the *tox*A gene, were found to be regularly distributed among *P. multocida* isolates. The results of PCR using fimbriae and adhesins encoding gene (*nan*B) and outer membrane proteins encoding gene (*omp*87) are supported by those reported by Katsuda et al. [11], who analyzed 378 *P. multocida* isolates using PCR and detected the presence of *nan*B and *omp*87 in most of the isolates. In addition, Jamali et al. [10] examined 141 *P. multocida* isolates for the detection of different virulence genes and found that *omp*87 and *nan*B genes were present in all isolates. On the other hand, the obtained PCR results of *M. haemolytica* are similar to those recorded by Singh et al. [14], who recorded *lkt* as species-specific for ruminants. In addition, Klima et al. [15] detected *lkt*C and *gcp* in all *M. haemolytica* isolates. Meanwhile, Klima et al. [42] recorded OMPs serine protease encoding (*ssa*) gene as one of the top ten antigens detected among 240 *M. haemolytica*. Moreover, Ayalew et al. [43] previously identified *M. haemolytica* OMPs that may be an important immunogen, including serotype 1-specific antigen (*ssa*1) by using immunoproteomic analyses.

In the present study, the *tox*A gene of *P. multocida* serotype B:2 and *lkt*C gene of *M. haemolytica* serotype 2 (the most prevalent serotype) were sequenced. Regarding *P. multocida tox*A gene, amino acid and nucleotide sequence analysis showed great homology between the Egyptian strain and the fourteen *P. multocida* strains uploaded in gene bank representing different clinical lesions, hosts, and localities worldwide. Generally, the sequence identities between the isolated Egyptian strain and different *P. multocida* strains uploaded from gene bank revealed 99.4–100% homology. This could suggest the high pathogenicity of the isolated strain and its high affinity to cause respiratory problems in infected animals [44], where *tox*A plays an important in destruction of lung tissues, and/or stimulation of a noxious host inflammatory response. Therefore, the *tox*A gene is considered an epidemiological marker found mostly in pneumonic *P. multocida* isolates [12]. Pullinger et al. [45] reported that the *P. multocida* toxin (PMT) acts as a potent mitogen. Sequence analysis of the structural gene for PMT, *tox*A, suggested that it was horizontally acquired because it had a low G+C content relative to the *P. multocida* genome [45]. Concerning *M. haemolytica lkt*C gene, amino acid and nucleotide sequence analysis also indicated great homology between the Egyptian strain and the seventeen *M. haemolytica* strains uploaded in genebank representing different clinical lesions, hosts, and localities worldwide. Sequence identities between the isolated Egyptian strain and different *M. haemolytica* strains uploaded in genebank revealed 92.6–100% homology. This leukotoxin has been implicated as a major virulence factor in the pathogenesis of *M. haemolytica*, helping in the colonization and invasion of the lung tissues by impairing the primary lung defense mechanism and subsequent immune response or by the induction of inflammation as a consequence of leukocyte lysis [46]. Therefore, characterization of such a gene provides important information about the pathogenicity of *M. haemolytica* [14]. Moreover, Highlander et al. [47] reported that *M. haemolytica* secreted a 102-kilodalton leukotoxin that was believed to be involved in the pathogenesis of severe bovine pneumonia and considered *lkt*C as the activator for leukotoxin (*lkt*A).

## 5. Conclusions

The situation of *P. multocida* and *M. haemolytica* infections in North Upper Egypt is complicated due to the different stresses facing the young calves, including managemental, environmental, and infectious factors. To control such infections, firstly, stress factors should be avoided in addition to rapid diagnosis and efficient treatment of disease calves using suitable antimicrobial agents after application of the sensitivity test. Detection of *Pasteurella* species in the clinical material was greatly accelerated by the use of molecular techniques such as PCR using universal genes. Serotyping of isolates indicated that *P. multocida* serotype B:2 and *M. haemolytica* serotype 2 were the most prevalent in Egypt. Detection of some virulence-associated genes indicated that all the tested *P. multocida* isolates harbored *nan*B, omp87, and *tox*A virulence genes, while *ssa*, *gcp*, and *lkt*C virulence genes were determined in most of tested *M. haemolytica* isolates. Sequencing of *P. multocida tox*A gene and *M. haemolytica lkt*C gene from the isolated strains indicated a great homology between the isolated strains and the other strains uploaded in gene bank of different clinical lesions, hosts and localities worldwide. More scientific and field benefits could be gained by further investigations to know more about the current epidemiological situation of *P. multocida* and *M. haemolytica* infections in Egypt, and this step is the cornerstone for the prevention and control of these diseases through designing autogenous vaccines or minimizing the importation of animals from the endemic countries.

## Figures and Tables

**Table 1:** Samples and bacterial isolates in different Governorates.

Governorates	No. of Samples	No. of Isolates		Total
		*P. multocida*	*M. haemolytica*	
El-Fayoum	106	22	10	32
Beni-Suef	83	11	5	16
Total	189	33	15	48

**Table 2:** Primers sequences and amplified products for the targeted genes for *P. multocida* and *M. haemolytica* isolates.

Bacteria	Target Gene		Primer Sequence 5′-3′	Amplified Product	Reference
*P. multocida*	*kmt*1	F	ATCCGCTATTTACCCAGTGG	460 bp	[18]
		R	GCTGTAAACGAACTCGCCAC		
	*nan*B	F	GTCCTATAAAGTGACGCCGA	554 bp	[19]
		R	ACAGCAAAGGAAGACTGTCC		
	*omp*87	F	AGGTGAAAGAGGTT ATG	200 bp	[20]
		R	TACCTAA CTCAACCAAC		
	*tox*A	F	CTTAGATGAGCGACAAGG	864 bp	[21]
		R	GAATGCCACACCTCTATAG		
*M. haemolytica*	*rpt*2	F	GTTTGTAAGATATCCCATTT	1022 bp	[22]
		R	CGTTTTCCACTTGCGTGA		
	*ssa*	F	TTCACATCTTCATCCTC	325 bp	[23]
		R	TTTTCATCCTCTTCGTC		
	*gcp*	F	CGCCCCTTTTGGTTTTCTAA	420 bp	[15]
		R	GTAAATGCCCTTCCATATGG		
	*lkt*C	F	GGAAACATTACTTGGCTATGG	440 bp	
		R	TGTTGCCAGCTCTTCTTGATA		

**Table 3:** Cycling conditions of the different primers during PCR.

Bacteria	Target Gene	Primary Denaturation	Secondary Denaturation	Amplification (35 Cycles)		
				Annealing	Extension	Final Extension
***P. multocida***	***kmt1***	94 C/5 min	94 C/30 s	55 C/1 min	72 C/1 min	72 C/10 min
	***nanB***	94 C/5 min	94 C/30 s	50 C/40 s	72 C/40 s	72 C/10 min
	***omp87***	94 C/5 min	94 C/30 s	48 C/30 s	72 C/30 s	72 C/7 min
	***toxA***	94 C/5 min	94 C/30 s	48 C/40 s	72 C/45 s	72 C/10 min
***M. haemolytica***	***rpt2***	95 C/3 min	95 C/1 min	48 C/1 min	72 C/30 s	72 C/10 min
	***ssa***	94 C/5 min	94 C/30 s	45 C/40 s	72 C/40 s	72 C/10 min
	***gcp***	94 C/5 min	94 C/30 s	58 C/40 s	72 C/40 s	72 C/10 min
	***lktC***	94 C/5 min	94 C/30 s	58 C/40 s	72 C/40 s	72 C/10 min

**Table 4:** Results of PCR as a confirmatory identification of *P. multocida* and *M. haemolytica* isolates.

Tested Bacteria	No. of the Tested Isolates	Positive PCR Result	
		No.	%
*P. multocida*	33	29	87.9
*M. haemolytica*	15	15	100

%: Percentages were calculated according to the corresponding number of the tested isolates.

**Table 5:** Prevalence of virulence-associated genes among the examined *P. multocida and M. haemolytica* isolates.

Tested Bacteria	Tested Gene	No. of the Tested Isolates	Positive		Negative	
			No.	%	No.	%
***P. multocida***	*nan*B	5	5	100	0	0
	*omp*87		5	100	0	0
	*tox*A		5	100	0	0
***M. haemolytica***	*ssa*	5	3	60	2	40
	*gcp*		4	80	1	20
	*lkt*C		4	80	1	20

%: Percentages were calculated according to the corresponding number of the tested isolates.

**Table 6:** Nucleotides and amino acid identity and sequence distance of *P. multocida* serotype B2 *tox*A gene between the isolated Egyptian strain and different strains uploaded in the gene bank.

Percent Identity																		
**Divergence**		**1**	**2**	**3**	**4**	**5**	**6**	**7**	**8**	**9**	**10**	**11**	**12**	**13**	**14**	**15**		
	**1**		99.8	99.8	99.8	99.9	99.9	99.9	99.9	99.9	99.8	99.9	99.8	99.6	99.5	99.9	**1**	AF240778.1
	**2**	0.1		99.8	99.8	99.9	99.9	99.9	99.9	99.9	99.8	99.9	99.8	99.6	99.5	99.9	**2**	EU873316.1
	**3**	0.1	0.2		99.8	99.9	99.9	99.9	99.9	99.9	99.8	99.9	99.8	99.6	99.5	99.9	**3**	EU849118.1
	**4**	0.1	0.2	0.2		99.9	99.9	99.9	99.9	99.9	99.8	99.9	99.8	99.6	99.5	99.9	**4**	AY965266.1
	**5**	0.0	0.1	0.1	0.1		100	100	100	100	99.9	100	99.9	99.8	99.6	100	**5**	CP003313.1
	**6**	0.0	0.1	0.1	0.1	0		100	100	100	99.9	100	99.9	99.8	99.6	100	**6**	FN398148.1
	**7**	0.0	0.1	0.1	0.1	0	0		100	100	99.9	100	99.9	99.8	99.6	100	**7**	FN398147.1
	**8**	0.0	0.1	0.1	0.1	0	0	0		100	99.9	100	99.9	99.8	99.6	100	**8**	EF441531.1
	**9**	0.0	0.1	0.1	0.1	0	0	0	0		99.9	100	99.9	99.8	99.6	100	**9**	AY854768.1
	**10**	0.0	0.1	0.1	0.1	0	0	0	0	0		99.9	99.8	99.6	99.5	99.9	**10**	AJ566110.1
	**11**	0.0	0.1	0.1	0.1	0	0	0	0	0	0		99.9	99.8	99.6	100	**11**	X57775.1
	**12**	0.1	0.2	0.2	0.2	0.1	0.1	0.1	0.1	0.1	0.1	0.1		99.6	99.5	99.9	**12**	X52478.1
	**13**	0.2	0.4	0.4	0.4	0.2	0.2	0.2	0.2	0.2	0.2	0.2	0.4		99.4	99.8	**13**	X51512.1
	**14**	0.4	0.5	0.5	0.5	0.4	0.4	0.4	0.4	0.4	0.4	0.4	0.5	0.6		99.6	**14**	EU849117.1
	**15**	0.0	0.1	0.1	0.1	0	0	0	0	0	0	0	0.1	0.2	0.4		**15**	**Egyptian Strain**
		**1**	**2**	**3**	**4**	**5**	**6**	**7**	**8**	**9**	**10**	**11**	**12**	**13**	**14**	**15**		

**Table 7:** Nucleotides and amino acid identity and sequence distance of *M. haemolytica* serotype A2 *lkt*C gene between the Egyptian strain and different strains uploaded in the genebank.

**Divergence**	**Percent Identity**																				
		**1**	**2**	**3**	**4**	**5**	**6**	**7**	**8**	**9**	**10**	**11**	**12**	**13**	**14**	**15**	**16**	**17**	**18**		
	**1**		100	100	100	100	100	100	100	100	100	100	100	100	95.7	95.7	94.6	92.6	100	**1**	CP023043.1
	**2**	0.0		100	100	100	100	100	100	100	100	100	100	100	95.7	95.7	94.6	92.6	100	**2**	CP023047.1
	**3**	0.0	0.0		100	100	100	100	100	100	100	100	100	100	95.7	95.7	94.6	92.6	100	**3**	CP023046.1
	**4**	0.0	0.0	0.0		100	100	100	100	100	100	100	100	100	95.7	95.7	94.6	92.6	100	**4**	CP023044.1
	**5**	0.0	0.0	0.0	0.0		100	100	100	100	100	100	100	100	95.7	95.7	94.6	92.6	100	**5**	CP004753.2
	**6**	0.0	0.0	0.0	0.0	0.0		100	100	100	100	100	100	100	95.7	95.7	94.6	92.6	100	**6**	CP004752.2
	**7**	0.0	0.0	0.0	0.0	0.0	0.0		100	100	100	100	100	100	95.7	95.7	94.6	92.6	100	**7**	CP011098.1
	**8**	0.0	0.0	0.0	0.0	0.0	0.0	0.0		100	100	100	100	100	95.7	95.7	94.6	92.6	100	**8**	CP005619.1
	**9**	0.0	0.0	0.0	0.0	0.0	0.0	0.0	0.0		100	100	100	100	95.7	95.7	94.6	92.6	100	**9**	CP005972.1
	**10**	0.0	0.0	0.0	0.0	0.0	0.0	0.0	0.0	0.0		100	100	100	95.7	95.7	94.6	92.6	100	**10**	CP005574.1
	**11**	0.0	0.0	0.0	0.0	0.0	0.0	0.0	0.0	0.0	0.0		100	100	95.7	95.7	94.6	92.6	100	**11**	CP005383.1
	**12**	0.0	0.0	0.0	0.0	0.0	0.0	0.0	0.0	0.0	0.0	0.0		100	95.7	95.7	94.6	92.6	100	**12**	M24197.1
	**13**	0.0	0.0	0.0	0.0	0.0	0.0	0.0	0.0	0.0	0.0	0.0	0.0		95.7	95.7	94.6	92.6	100	**13**	M20730.1
	**14**	4.4	4.4	4.4	4.4	4.4	4.4	4.4	4.4	4.4	4.4	4.4	4.4	4.4		100	98	93.1	95.7	**14**	CP006957.2
	**15**	4.4	4.4	4.4	4.4	4.4	4.4	4.4	4.4	4.4	4.4	4.4	4.4	4.4	0.0		98	93.1	95.7	**15**	KT013279.1
	**16**	5.6	5.6	5.6	5.6	5.6	5.6	5.6	5.6	5.6	5.6	5.6	5.6	5.6	2.1	2.1		94.6	94.6	**16**	CP006573.1
	**17**	7.9	7.9	7.9	7.9	7.9	7.9	7.9	7.9	7.9	7.9	7.9	7.9	7.9	7.4	7.4	5.6		92.6	**17**	U01215.1
	**18**	0.0	0.0	0.0	0.0	0.0	0.0	0.0	0.0	0.0	0.0	0.0	0.0	0.0	4.4	4.4	5.6	7.9		**18**	**Egyptian strain**
		**1**	**2**	**3**	**4**	**5**	**6**	**7**	**8**	**9**	**10**	**11**	**12**	**13**	**14**	**15**	**16**	**17**	**18**		
